# m6A regulator‐mediated RNA methylation modification patterns and immune microenvironment infiltration characterization in severe asthma

**DOI:** 10.1111/jcmm.16961

**Published:** 2021-10-14

**Authors:** Deyang Sun, Huan Yang, Liming Fan, Fenglin Shen, Zhen Wang

**Affiliations:** ^1^ The First Clinical College Zhejiang Chinese Medical University Hangzhou China; ^2^ Department of Respiration The First Affiliated Hospital of Zhejiang Chinese Medical University Hangzhou China

**Keywords:** epigenetics, immune microenvironment, m6A, m6A modification pattern, severe asthma

## Abstract

N6‐methyladenosine (m6A) modification is one of the most prevalent RNA modification forms of eukaryotic mRNA and is an important post‐transcriptional mechanism for regulating genes. However, the role of m6A modification in the regulation of severe asthma has never been reported. Thus, we aimed to investigate the m6A regulator‐mediated RNA methylation modification patterns and immune microenvironment infiltration characterization in severe asthma. In this study, 87 healthy controls and 344 severe asthma cases from the U‐BIOPRED (Unbiased Biomarkers for the Prediction of Respiratory Disease Outcomes) programme were used to systematically evaluate the m6A modification patterns mediated by 27 m6A regulators and to investigate the effects of m6A modification on immune microenvironment characteristics. We found that 16 m6A regulators were abnormal and identified two key m6A regulators (YTHDF3 and YTHDC1) and three m6A modification patterns. The study of infiltration characteristics of immune microenvironment found that pattern 2 had more infiltrating immune cells and more active immune response. Besides, it was found that the eosinophils which are very important for severe asthma were affected by YTHDF3 and EIF3B. We also verified key m6A regulators with merip‐seq and found that they were mainly distributed in exons and enriched in 3′UTR. In conclusion, our findings suggested that m6A modification plays a key role in severe asthma, and may be able to guide the future strategy of immunotherapy.

## 1 INTRODUCTION

Asthma is a chronic airway inflammatory disease with obvious heterogeneity and complex pathophysiological manifestations.^1^ At present, there are at least 300 million cases of asthma in the world, and the prevalence of asthma is increasing year by year.^2^ Severe asthma refers to the severe state of asthma. Although severe asthma accounts for a small proportion of the total number of asthma, its direct medical costs account for 50% of the total cost of asthma treatment.^3^ Severe asthma is difficult to control, which seriously affects the quality of life of patients. Besides, its frequent attacks lead to high medical costs, which has become a serious social and economic burden, and also the main cause of disability and death of asthma.^4^ Therefore, to improve the understanding of the pathogenesis of severe asthma is important to ameliorate its attack and prognosis, as well as to reduce medical costs. Furthermore, it has been found that the incidence of severe asthma is familial aggregation, and there are multiple susceptibility genes. The incidence of people with susceptibility genes is greatly affected by environmental factors.^5^ So, in‐depth study of gene‐environment interaction will help to reveal the genetic mechanism of severe asthma.

As one of the most important discoveries and research hotspots in recent years, epigenetics plays an important role in elucidating the interaction between genes and environment and changing the course of disease. It provides instructions for when, where and how to apply genetic information.^6^ The in‐depth study on the epigenetic mechanism of severe asthma is conducive to investigating the relationship between genes and environmental factors, and to formulating effective treatment strategies for severe asthma.^7^ Epigenetics mainly includes DNA methylation, RNA modification and histone modification. N6‐methyladenosine (m6A) modification is the most prevalent form of RNA modification in eukaryotic mRNA and even viral RNA.^8^, ^9^ M6A modification has been reported since the 1970s, but the overall distribution of the modification in RNA and its effect on gene expression regulation have been poorly understood. In 2011, the first real RNA demethylase fat mass‐and obesity‐associated gene (FTO) was reported, and the methylation modification of m6A was proved to be reversible, which made the study of mRNA methylation come into the eyes of scientists again.^10^ The regulatory proteins of m6A were composed of methyltransferases (writers), demethylases (erasers) and methylated reading proteins (readers).^11^ Methyltransferases include methyltransferase‐like 3 (METTL3), methyltransferase‐like 3 (METTL4), RNA‐binding motif protein15 (RBM15), Cbl proto‐oncogene‐like 1 (CBLL1), WT1‐associated protein (WTAP) and so on. Their main function is to catalyse the m6A modification of mRNA.^12^ On the contrary, the demethylases is to demethylation of bases that have undergone m6A modification, it includes FTO and Human AlkB homolog H5 (ALKBH5).^13^ Methylated reading proteins are mainly proteins of the YT521‐B homology (YTH) domain family, including YTH Domain Containing 1(YTHDC1), YTH Domain Containing 2(YTHDC2), YTH m6A RNA‐binding protein 1 (YTHDF1), YTH m6A RNA‐binding protein 2 (YTHDF2) and YTH m6A RNA‐binding protein 3 (YTHDF3); its main function is to identify the bases that undergo m6A modification and thus activate downstream regulatory pathways such as RNA degradation and miRNA processing.^14^

Abnormalities of these regulators can affect mRNA in many aspects, including structure, splicing, translation, stability and so on, leading to the occurrence of disease.^15^ It can even explain the potential mechanism of immune regulation in some diseases. Although more and more evidence shows the regulatory role of m6A in immune response^16^ and also proves the diagnostic significance of m6A regulatory pattern in childhood asthma,^17^ there is still a gap in the research on severe asthma and m6A. Therefore, In this study, we systematically evaluated the m6A regulator‐mediated RNA methylation modification patterns and immune microenvironment infiltration characterization in severe asthma.

## 2 METHODS

### 2.1 Data download and processing

Data for this study were from U‐BIOPRED^18^ (Unbiased Biomarkers for the Prediction of Respiratory Disease Outcomes), a multicenter prospective cohort study involving 16 clinical centres in 11 European countries, downloaded from Gene Expression Omnibus data sets (https://www.ncbi.nlm.nih.gov/geo/). The serial number is GSE69683, and the sample type is blood. A total of 87 healthy controls and 344 severe asthma cases were selected. The platform was GPL13158 [HT_HG‐U133_Plus_PM] Affymetrix HT HG‐U133+ PM Array Plate. R software and annotation package were used to obtain the gene symbols of the dataset.

### 2.2 Selection and function prediction of m6A regulators

Twenty‐seven widely recognized m6A RNA methylation regulators were selected from published literatures. It includes CBLL1, methyltransferase‐like 14 (METTL14), METTL3, METTL4, zinc finger CCCH domain‐containing protein 13 (ZC3H13), zinc Finger Protein 217 (ZNF217), RNA‐binding motif protein15B (RBM15B), WTAP, RBM15, KIAA1429, YTHDF2, eukaryotic translation initiation factor 3 subunit A (EIF3A), eukaryotic translation initiation factor 3 subunit B (EIF3B), HNRNPA2B1, heterogeneous nuclear ribonucleoprotein C (HNRNPC), insulin‐like growth factor 2 mRNA‐binding protein 1 (IGF2BP1), insulin‐like growth factor 2 mRNA‐binding protein 2 (IGF2BP2), insulin‐like growth factor 2 mRNA‐binding protein 3 (IGF2BP3), YTHDC1, YTHDC2, YTHDF1, YTHDF3, leucine‐rich pentatricopeptide repeat containing (LRPPRC), FMRP translational regulator 1 (FMR1), ELAV‐like RNA‐binding protein 1 (ELAVL1), ALKBH5 and FTO.^19^, ^20^, ^21^, ^22^, ^23^ The protein‐protein interaction networks of 27 m6A regulators were obtained from string database (https://string‐db.org/) and processed with Cytoscape.

### 2.3 Difference analysis of m6A regulators

The differential expression of 27 m6A RNA methylation regulators between severe asthma cases and healthy controls was analysed by Wilcox.test and the up/down/unchanged genes were visualized with R package ‘ggplot2’. The R package ‘corrplot’ was used to identify the correlation between the methylation regulators. The potential m6A RNA methylation regulators in patients with severe asthma were identified by univariate logistic regression and were cut off by *p* < 0.05. The least absolute shrinkage and selection operator (LASSO) Cox regression was used for feature selection and dimension reduction, and the risk scores of potential severe asthma related genes were calculated for verification. Receiver operating characteristic (ROC) curve analysis was used to evaluate the distinguishing performance.

### 2.4 Non‐negative matrix factorization consensus clustering

According to the expression of 27 m6A regulators, Non‐negative matrix factorization (NMF) was performed to identify different m6A modification patterns. NMF was to identify potential features in gene expression profile by resolving the original matrix into k non‐negative matrices.^24^ The deposition was repeated, and the results were aggregated to obtain consistent clustering. According to the co‐occurrence coefficient, dispersion and profile coefficient, the most suitable number of subtypes was determined. NMF was performed by an R package called ‘NMF’. Principal component analysis (PCA) was used to further verify the expression patterns of 27 m6A regulators in different modification patterns.

### 2.5 Biological function analysis of m6A modification patterns

In order to study the biological functions of genes in m6A modification patterns, we compared their Kyoto Encyclopedia of Genes (KEGG) pathway enrichment and gene ontology (GO) analysis, and analysed their biological functions by Gene Set Enrichment Analysis (GSEA) with R ‘clusterprofiler’ package.^25^ In short, n random gene sets of size k were randomly sampled in a straightforward way. The ‘ont’ of gsego was set to ‘all’, which includes molecular function (MF), biological process (BP) and cellular component (CC) categories. All terms were ranked according to the enrichment score, and the top four results were taken.

### 2.6 Analysis of infiltration characteristics of immune microenvironment

Single‐sample gene‐set enrichment analysis (ssgsea) was used to estimate the number and reactivity of specific infiltrating immune cells, which defined an enrichment score by calculating the enrichment degree of samples in a given data set. Wilcox test was used to compare the enrichment scores among the groups. HLA is important in immune microenvironment,^23^ so we also analysed the association between HLA‐related genes and m6A regulators. The correlation of m6A regulators with immune reaction activity and HLA gene expression was determined by Spearman correlation analysis with R package ‘corrplot’.

### 2.7 Identification of genes mediated by m6A regulators

The R software package ‘WGCNA’ was used to construct co‐expression module networks and to identify the genes mediated by m6A regulators. In briefly, there were two steps.^26^ The first step was to calculate the weighted value of the correlation coefficient, that is, to take the n‐th power of the gene correlation coefficient to construct a scale‐free network. In the second step, hierarchical clustering tree was constructed by Person Coefficient. Based on the weighted correlation coefficient of genes, genes were classified according to expression patterns, and the adjacency matrix was transformed into topological overlap matrix (TOM). Through dynamic tree cutting, modules were formed by the differences based on TOM. Here, we set the minimum module size to 100 and the cutting height to 0.25. Finally, the correlation between key m6A regulators and related modules was calculated, and the related module was enriched by Kyoto Encyclopedia of Genes (KEGG) pathway and analysed by Gene Ontology (GO) using The Database for Annotation, Visualization and Integrated Discovery (DAVID) v6.8; the enrichment pathway was cut off by *p* < 0.05.

### 2.8 Mouse model and determination of m6A methylation peak

We modelled two groups of female 6‐week‐old BALB/C mice^27^: severe asthma group and blank control group (*n* = 3 per group). They had the same feeding conditions and growth environment. Immunization solution: Dissolve 20 mg ovalbumin (OVA) in 1 ml normal saline (NS), after OVA is completely dissolved, dilute 0.4–10 ml and mix well, then it was mixed with the same volume of liquid aluminium adjuvant and placed on a shaking table at 4℃ for 30 min. Challenge solution: Add 0.5 g OVA into 10 ml NS, fully dissolve it, and shake it on a shaking table at 4℃ for 30 min. Immunization: Mice were injected intraperitoneally on days 0 and 12, each with 0.2 ml; the control group was treated with equal volume of normal saline. Challenge: On days 18–23, the mice were atomized by ultrasound in a closed container at a dose of 10 ml once a day for 20 min. Lung tissue was taken 24 h after the last atomization and immediately stored in liquid nitrogen. All experimental procedures used in this study were approved and conducted according to the guidelines by the laboratory Animal Management Committee of Zhejiang Chinese Medical University. RNA isolation and methylated RNA immunoprecipitation sequencing (Merip‐seq) was completed by Beijing Genomics Institute. According to the TDF file of sequencing samples, the m6A methylation peak was displayed by IGV software.

## 3 RESULTS

### 3.1 The landscape of selected m6A regulators and its abnormal expression in severe asthma

**FIGURE 1:**
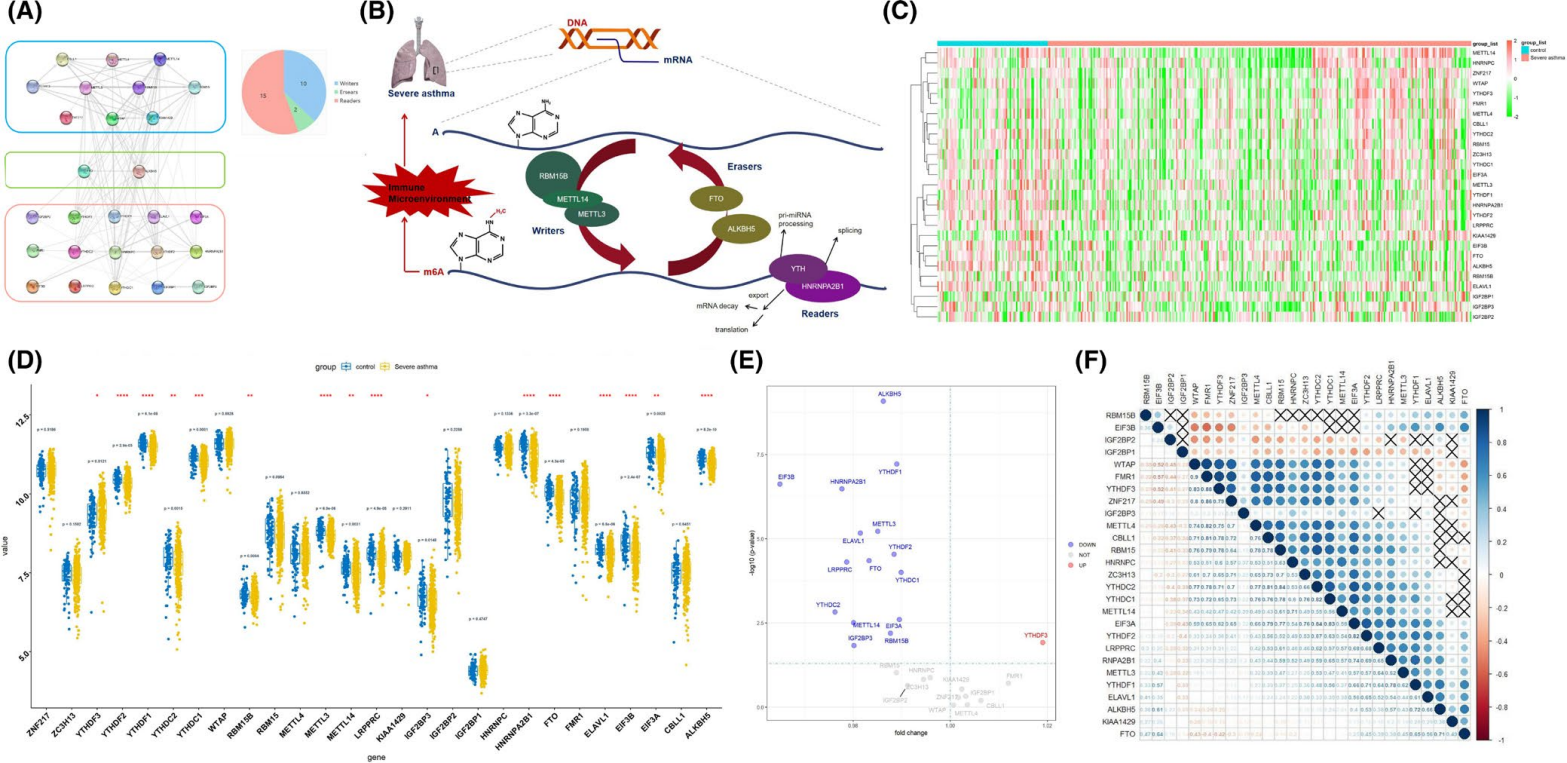
The landscape of selected m6A regulators and its abnormal expression in severe asthma. (A) Component analysis of the m6A regulator and protein‐protein interaction among 27 m6A RNA methylation regulators. (B) The overview of the dynamic reversible process of m6A RNA methylation modification in severe asthma. (C and D) The heat map and box plot demonstrated the expression differences of 27 m6A RNA methylation regulators between severe asthma cases and healthy controls. (E) Volcano map of the expression differences of 27 m6A RNA methylation regulators between healthy controls and severe asthma samples. (F) Correlations among the expression of 27 m6A regulators in all samples, *p *> 0.05 was marked as ‘×’

The 27 selected m6A regulators included 10 writers (CBLL1, METTL14, METTL3, METTL4, ZC3H13, ZNF217, RBM15B, WTAP, RBM15 and KIAA1429), 15 readers (YTHDF2, EIF3A, EIF3B, HNRNPA2B1, HNRNPC, IGF2BP1, IGF2BP2, IGF2BP3, YTHDC1, YTHDC2, YTHDF1, YTHDF3, LRPPRC, FMR1 and ELAVL1) and 2 erasers (ALKBH5 and FTO). In order to determine the relationship between the 27 m6A RNA methylation regulators, PPI network analysis was carried out. The results showed that the 27 regulators were closely related and the writers, readers and erasers usually function as a complex (Figure 1A). Besides, its function on immune microenvironment of severe asthma was also reviewed (Figure 1B). By analysed the expression differences of 27 m6A RNA methylation regulators between severe asthma cases and healthy controls, 16 m6A RNA methylation regulators were found to be significantly associated with the occurrence of severe asthma (Figure 1C,D). Compared with healthy controls, there were 15 downregulated genes and 1 up‐regulated gene in severe asthma cases (Figure 1E). Downregulated genes included three writers (METTL14, METTL3 and RBM15B), ten readers (YTHDF2, EIF3A, EIF3B, HNRNPA2B1, IGF2BP3, YTHDC1, YTHDC2, YTHDF1, LRPPRC and ELAVL1) and two erasers (ALKBH5 and FTO). The up‐regulated gene was YTHDF3. At the same time, the correlation of these 27 regulators was analysed. The results showed that most of the regulators in the sample were significantly correlated (*p *< 0.05), and the most significant positive correlations were EIF3A‐YTHDC2 and RBM15‐YTHDC2, with correlation *R* values of 0.84. The most significant negative correlation was FMR1‐ EIF3B, with correlation *R* values of 0.57 (Figure 1F).

### 3.2 Identification of crucial m6A regulators in severe asthma

**FIGURE 2:**
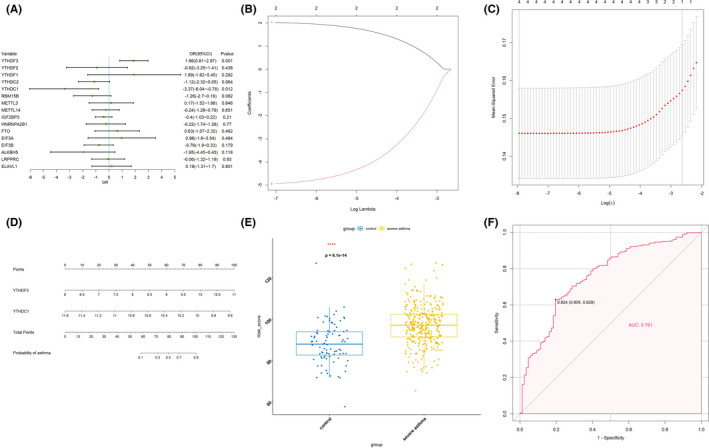
Identification of crucial m6A regulators in severe asthma. (A) Univariate logistic regression investigated the relationship between m6A regulators and severe asthma cases, revealing two severe asthma related m6A regulators (*p* < 0.05). (B) Least absolute shrinkage and selection operator (LASSO) coefficient profiles of 2 severe asthma related m6A regulators. (C) 10‐fold cross‐validation for tuning parameter selection in the LASSO regression. (D) The risk scores of 2 severe asthma related m6A regulators. (E) The risk distribution between healthy and severe asthma cases, where severe asthma cases have a much higher risk score than healthy controls. (F) The discrimination ability for healthy and severe asthma cases by m6A regulators was analysed by ROC curve and evaluates by AUC value

**FIGURE 3:**
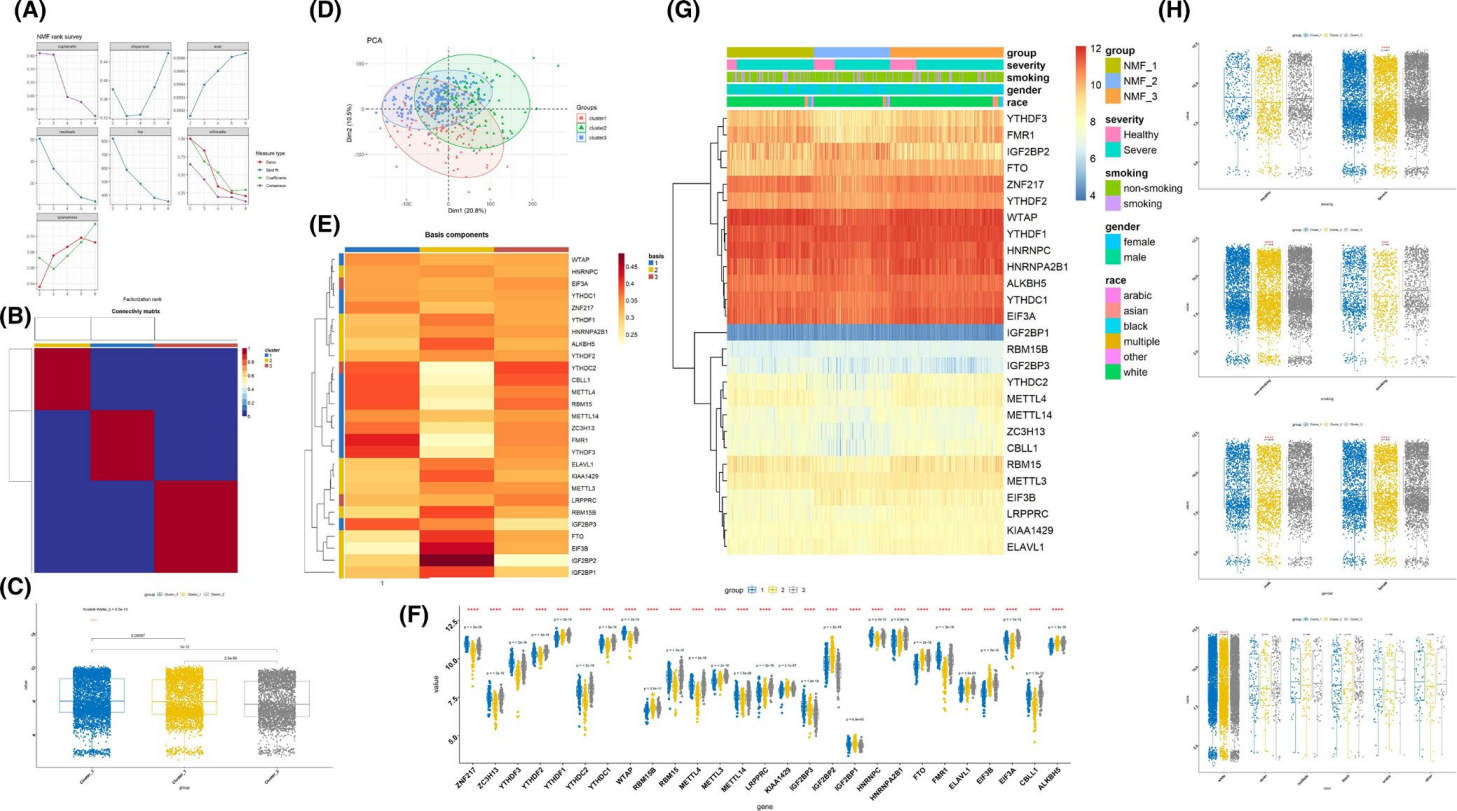
Three patterns of m6A RNA methylation mediated by 27 regulators. (A and B) Evaluation model for k = 2–6, according to the copynetic value, the best rank is 3. (C) Box plot of samples under three kinds of m6A RNA methylation regulation patterns. (D) Principal component analysis of samples under three kinds of m6A RNA methylation regulation patterns. (E) Basismap of samples under three kinds of m6A RNA methylation regulation patterns. (F) Box plot of expression status of 27 m6A regulators under three kinds of m6A RNA methylation regulation patterns. (G) The correlation analysis of m6A methylation regulators and clinical phenotypes in three kinds of m6A RNA methylation regulation patterns. (H) The expression of three clusters in the groups of pathogenicity, smoking, gender and race

In order to investigate the m6A regulators’ contribution to the pathogenesis of severe asthma, a series of bioinformatics analysis were used. We examined the expression data of the 16 difference‐expressed genes using univariate logistic regression to identify the potential m6A regulators, and two regulators (YTHDF3 and YTHDC1) were found to be associated with severe asthma (Figure 2A). Next, LASSO Cox regression was used for feature selection and dimension reduction, and it was found that these two regulators were all important for severe asthma (Figure 2B,C). At the same time, the risk scores of the two regulators were compared between the severe asthma group and the healthy control group. It was found that the risk scores of the two regulators in the severe asthma group were significantly higher than that in the healthy control group (*p* = 6.1e‐14) (Figure 2D,E); ROC curve also illustrated the two m6A regulators possess a good performance in classifying patients with severe asthma and healthy controls (Figure 3F), which indicated that these two m6A regulators play a crucial role in severe asthma and can affect the prognosis of severe asthma.

### 3.3 Three patterns of m6A RNA methylation mediated by 27 regulators

According to the value of copynetic calculated by ‘NMF’ package, the optimal value of k is three (Figure 3A,B), that is, there were three different m6A RNA methylation patterns with qualitatively different expression of 27 m6A regulators, including 132 samples in cluster 1 (15 healthy samples and 117 severe asthma samples), 116 samples in cluster 2 (32 healthy samples and 84 severe asthma samples) and 173 samples in cluster 3 (40 healthy samples and 133 severe asthma samples). There were significant differences among the samples in each modification pattern (*p* < 0.001) (Figure 3C). Besides, Principal component analysis was also performed (Figure 3D). According to the basismap, we can clearly see the driving signatures in each pattern (Figure 3E). At the same time, the differences of 27 m6A regulators in each modification pattern were compared, and the results showed that all the regulators had significant differences (*p* < 0.001) (Figure 3F). We also found that the expression of these three clusters was different in the groups of smoking, gender and race, which indicated that the methylation of m6A RNA might be related to the clinical phenotype of severe asthma (Figure 3G,H).

### 3.4 Biological function analysis of three m6A modification patterns

**FIGURE 4:**
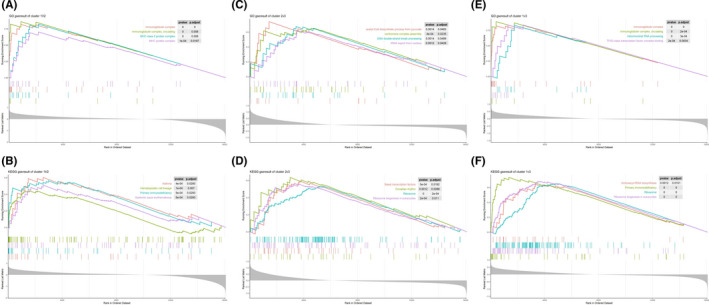
Biological function analysis of three m6A modification patterns. (A and B) Gene Set Enrichment Analysis (GSEA) of gene ontology (GO) analysis and Kyoto Encyclopedia of Genes (KEGG) pathway enrichment compared with cluster 1 and cluster 2. (C and D) Gene Set Enrichment Analysis (GSEA) of gene ontology (GO) analysis and Kyoto Encyclopedia of Genes (KEGG) pathway enrichment compared with cluster 2 and cluster 3. (E and F) Gene Set Enrichment Analysis (GSEA) of gene ontology (GO) analysis and Kyoto Encyclopedia of Genes (KEGG) pathway enrichment compared with cluster 1 and cluster 3

In order to understand the biological reactions in the three m6A modification patterns, we compared the GO analysis and KEGG pathway between them. Compared with cluster 1 and cluster 2, GO analysis results mainly involved in immune process. According to the enrichment score, the top four items were as follows: immunoglobulin complex, MHC class II protein complex, immunoglobulin complex and MHC protein complex (Figure 4A). The results of KEGG pathway include asthma, primary immunity, systemic lupus erythematosus and hematopoietic cell lineage (Figure 4B). The item of “asthma” ranks first, it is proved that pattern 1 and pattern 2 of m6A methylation modification may affect the immune progress of asthma. Compared with cluster 2 and cluster 3, GO analysis results showed that these genes could affect acetyl CoA biosynthetic process from pyramid, DNA double‐strand break processing, central complex assembly and tRNA export from nucleus (Figure 4C). KEGG pathway results showed that these genes could affect circadian rhythms, basic transcription factors, ribosome biogenesis in eukaryotes and ribosome (Figure 4D). It is suggested that the m6A methylation modification in pattern 2 and pattern 3 may affect the post‐transcriptional translation of RNA in severe asthma. Compared with cluster1 and cluster3, the results were still related to immunity. The first four items of GO analysis results were immunoglobulin complex, circulating, TFIID class transcription factor complex binding, mitochondrial RNA processing and immunoglobulin complex (Figure 4E). The results of KEGG pathway showed that the first four items were primary immunity, ribosome biogenesis in eukaryotes, ribosome and aminoacyl tRNA biosynthesis (Figure 4F).

### 3.5 Infiltration characteristics of immune microenvironment

**FIGURE 5:**
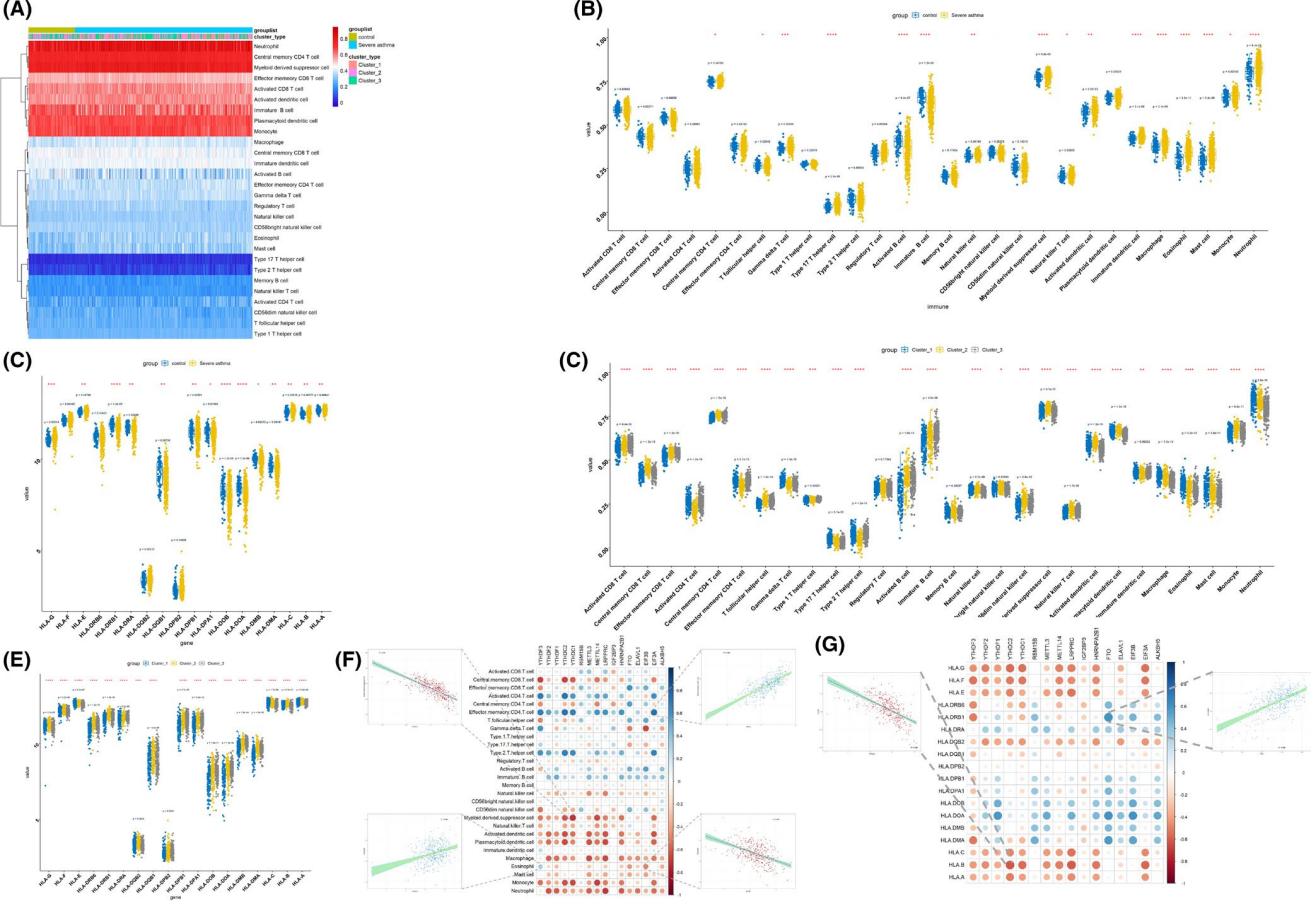
Immune microenvironment infiltration characterization. (A) Heat map of the abundance differences of each immune microenvironment infiltrating immunocyte in each group. (B and C) Box plot of the abundance differences of each immune microenvironment infiltrating immunocyte and HLA genes in healthy controls and severe asthma cases. (D and E) Box plot of the abundance differences of each immune microenvironment infiltrating immunocyte and HLA genes in 3 m6A modification patterns. (F) The correlation analysis of the m6A differentially expressed genes (GEGs). The most positive correlated immunocyte‐m6A regulator pair is EIF3A‐effector memory CD4 T cell, the most negative correlation was YTHDC1‐myeloid‐derived suppressor cell, and the fraction status is presented at up panel. The abundance of eosinophils is positively correlated with YTHDF3 and negatively correlated with EIF3B; the fraction status is presented at down panel. (G) The correlation between HLA genes and m6A DEGs

In order to investigate the differences of immune microenvironment characteristics between severe asthma and health control and between different m6A modification patterns, we evaluated the expression of infiltrating immune cell genes and HLA genes. The results showed that the infiltration level of infiltrating immune cells in severe asthma group was higher than that in healthy control group. Besides, the expression of most HLA genes had also changed, such as HLA‐DRB1, HLA‐DOB and HLA‐DOA (Figure 5A–C). Moreover, we found many immunocytes were different among 3 clusters. Compared with cluster 2 and cluster 3, cluster 1 had higher infiltration levels of gamma delta T cell, type 17 T helper cell, macrophage, eosinophil, mast cell and neutrophil, while cluster 2 had more infiltrating immune cells and more active immune response than cluster 1 and cluster 3, including central memory CD4 T cell, effector memory CD4 T cell, activated B cell, natural killer cell, CD56dim natural killer cell, myeloid‐derived suppressor cell, natural killer T cell, activated dendritic cell, plasmacytoid dendritic cell and monocyte. However, the infiltration level of cluster 3 was lower than that of cluster 1 and cluster 2, only activated CD8 T cell, central memory CD4 T cell, type 1 T helper cell and immature B cell had higher infiltration level (Figure 5A,D). The same trend was found in HLA gene expression (Figure 5E). Besides, the correlation analysis of the m6A differentially expressed genes (GEGs) showed that the m6A regulator was related to many immune cells, the most positive correlation was EIF3A and effector memory CD4 T cell, and the most negative correlation was YTHDC1 and myeloid‐derived suppressor cell (Figure 5F). We also found that many immune cells are co‐regulated by the m6A regulator, such as eosinophils, which are closely related to severe asthma, the abundance of eosinophils is positively correlated with YTHDF3 and negatively correlated with EIF3B, which proves that the expression of YTHDF3 and EIF3B has a close influence on eosinophils in the pathogenesis of severe asthma. Similarly, we also analysed the correlation between HLA genes and m6A DEGs, and found that HLA_DRB1‐FTO was the most positively correlated pair, the most negatively correlated HLA‐m6A pair was HLA_B‐YTHDC2 (Figure 5G).

### 3.6 Gene expression regulatory network mediated by m6A regulators

**FIGURE 6:**
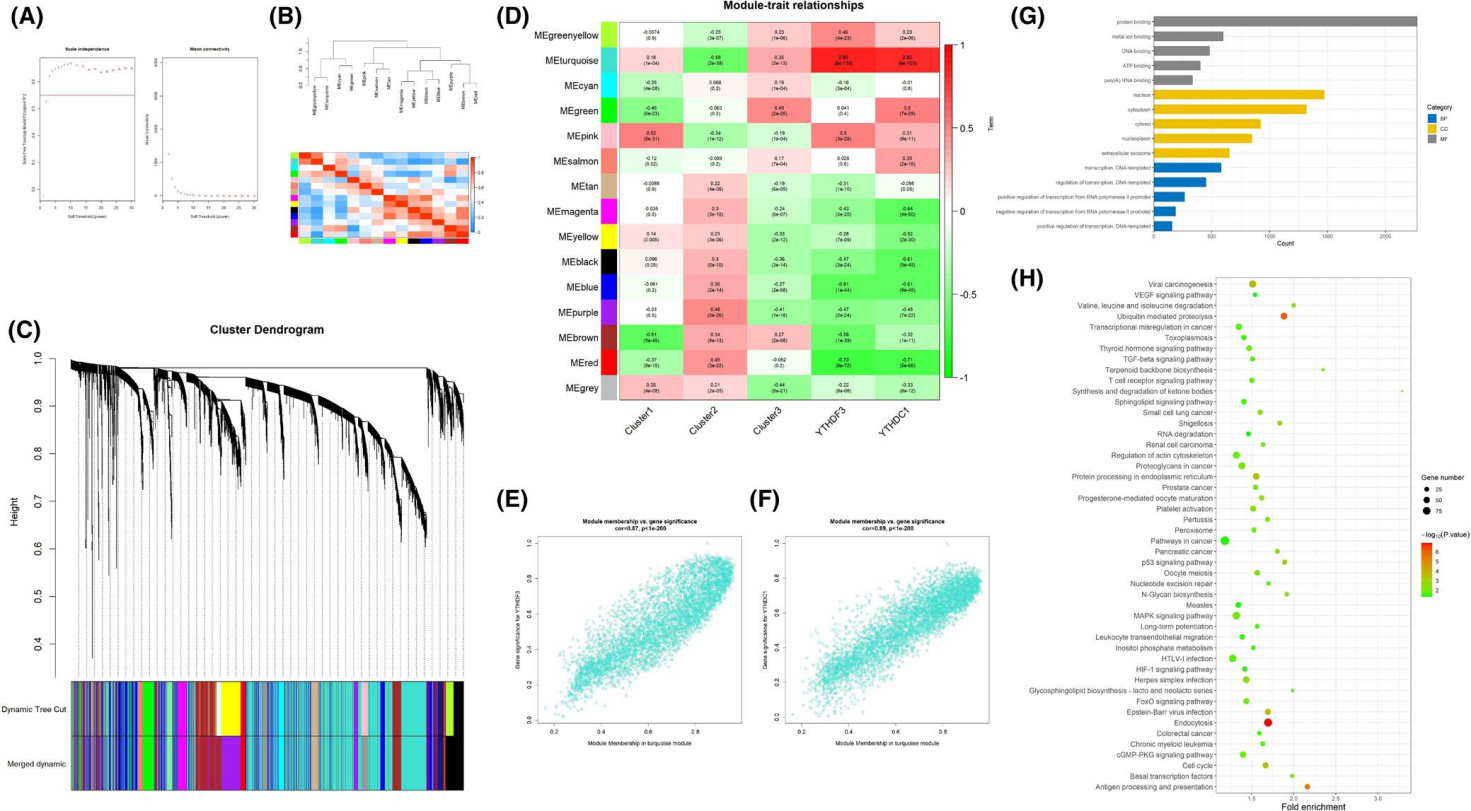
Gene expression regulatory network mediated by m6A regulators. (A) Analysis of the scale‐free ft index and analysis of the mean connectivity for various soft‐thresholding powers, and the power was four. (B) Correlation diagram between modules. (C) Hierarchical clustering tree to show each module. (D) The correlation of 14 gene modules with different m6A modification patterns and key m6A genes. (E and F) Module membership vs. gene significance for YTHDF3 and YTHDC1. (G and H) The biological function of genes in turquoise module

**FIGURE 7:**
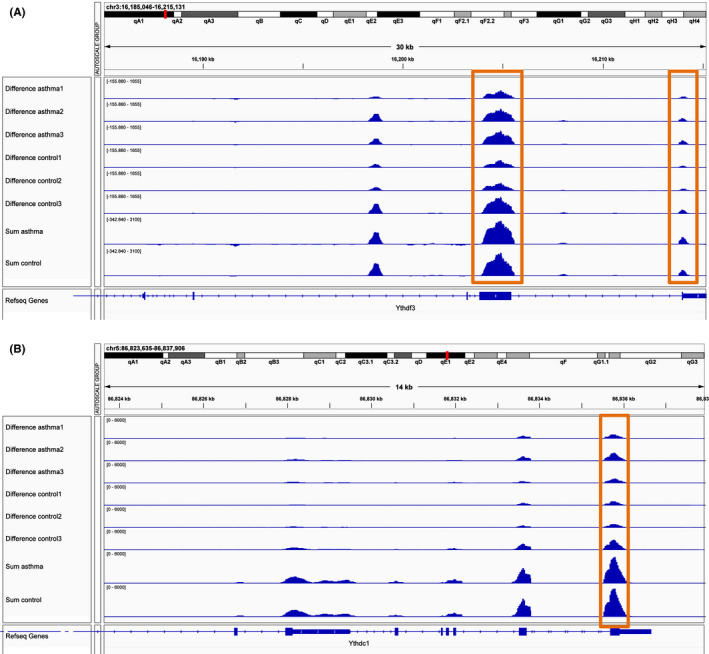
m6A methylation peaks of YTHDF3 and YTHDC1. (A) Integrative genomics viewer (IGV) plots showing m6A methylated peaks for YTHDF3. (B) IGV plots showing m6A methylated peaks for YTHDC1. Blue boxes represent exons, and blue lines represent introns

By weighted gene co‐expression network analysis (WGCNA), the weighted value of the correlation coefficient was calculated to be four, and twenty‐four gene modules were identified (Figure 6A–C). Next, we analysed the correlation of these twenty‐four gene modules with different m6A modification patterns and key m6A genes. The results showed that cluster 2 (*r* = −0.58, *p* = 2e‐38), YTHDF3 (*r* = 0.85, *p* = 8e‐119) and YTHDC1 (*r* = −0.82, *p* = 6e‐103) were closely related to the turquoise module (Figure 6D–F), so we analysed the biological function of genes in turquoise module (Figure 7G,H). Go analysis showed that these genes were involved in 194 biological processes (BP) (protein binding, metal binding, DNA binding, ATP binding, poly (a) RNA binding, etc.), 98 cell components (CC) (nucleus, cytochrome, cytosol, nucleoplasm, extracellular exosome, etc.) and 74 molecular functions (MF), (transcription DNA‐templated, regulation of transcription DNA‐templated, positive regulation of transcription from RNA polymerase II promoter, negative regulation of transcription from RNA polymerase II promoter, positive regulation of transcription DNA‐templated, etc.). KEGG enriched 48 items. These results suggest that these genes regulated by m6A may play an important role in severe asthma by affecting these biological processes, cell components, molecular functions and pathways.

### 3.7 m6A methylation peaks of YTHDF3 and YTHDC1

In order to verify the above results, we sequenced the whole methylated RNA species in the lung tissues of severe asthma mice and control mice using the previously described merip‐seq method.^28^ The methylation peaks of YTHDF3 and YTHDC1 were visualized according to the TDF file. It was found that the methylation peaks of key m6A regulators in severe asthma were different from those in the control group, and were mainly distributed in exons (Figure 7A,B). In addition, it was found that both YTHDF3 and YTHDC1 transcripts had m6A enrichment in 3′UTR (Figure 7A,B).

## 4 DISCUSSION

Asthma is a chronic inflammatory respiratory disease, which involves many inflammatory cells, immune cells and cell components.^1^ It affects about 300 million people around the world and is expected to increase by another one‐third by 2025. Severe asthma refers to the severe state of asthma, half of the cases are accompanied by chronic rhinosinusitis and nasal polyps, the condition of patient fluctuates, and acute exacerbation often occurs, which cannot be controlled by drugs.^4^ m6A RNA methylation plays an important role in regulating the expression of pathogenic genes; abnormal m6A modification can affect RNA splicing, translocation and translation, resulting in the occurrence of diseases. Recently, the Genotype‐Tissue Expression (GTEx) project reported 129 transcriptome‐wide m6A profiles, covering 91 individuals and 4 tissues (brain, lung, muscle and heart).^29^ For lung, 62 m6A quantitative trait loci (QTLs) colocalize with genome‐wide association studies (GWAS) variants. Asthma‐associated rs3194051 is a lung m6A QTL for immune‐related interleukin‐7 (IL‐7) that contributes to atopic asthma, acting in bronchoalveolar lavage fluid and regulating airway eosinophilia. The results provided important insights and resources for understanding the relationship between asthma and m6A. Moreover, in another study on childhood asthma found that m6A regulators also played a crucial role, and screened five candidate m6A regulators (FMR1, KIAA1429, WTAP, YTHDC2 and ZC3H13) to predict the risk of childhood asthma.^17^ However, there is still a blank in the field of severe asthma and m6A. Besides, recent studies have shown that m6A modification plays an important role in innate immune response and adaptive immune response,^30^, ^31^, ^32^ it has been proved that it plays an important role in tumour immune microenvironment,^33^, ^34^, ^35^ so we believe that in the pathogenesis of severe asthma, there are similar to the occurrence of m6A modification, and this was confirmed in this study. In this study, a series of analyses were carried out to elucidate the m6A regulator‐mediated RNA methylation modification patterns and immune microenvironment infection characterization in severe asthma.

First of all, we found that 16 m6A regulators were changed in severe asthma group compared with healthy control group, which indicated that m6A regulators were involved in the process of severe asthma. Through correlation analysis, it was found that they cooperated with each other significantly, which indicated that writers, readers and erasers jointly affected the occurrence and development of severe asthma. After a series of analysis such as univariate logistic regression and lasso Cox regression, it was found that YTHDF3 and YTHDC1 were more important among the 16 m6A regulators, which were also verified by risk score and merip‐seq, the results showed that the methylation peaks of YTHDF3 and YTHDC1 in severe asthma were different from those in the control group, and were mainly distributed in exons and enriched in 3′UTR. Secondly, we identified three m6A modification patterns, and 27 m6A regulators could distinguish each pattern well, which proves the importance of m6A regulator in severe asthma again. We also found that the expression of these three clusters was different in the groups of smoking, gender and race, which indicated that the methylation of m6A RNA might be related to the clinical phenotype of severe asthma. Thirdly, we studied the differences of immune microenvironment characteristics between severe asthma group and health control group and between different m6A modification patterns, the results showed that the immune response of severe asthma group was more active, and the pattern 2 had more infiltrating immune cells, while pattern 3 had the lowest immune response. According to the immune characteristics of different models, it can provide a theoretical basis for the classification of immune subtypes of severe asthma. This study can make us more in‐depth understand the regulatory mechanism of immune microenvironment of severe asthma, which is more conducive to the clinical precise treatment. Recently, a study used this method to divide gastric cancer into three kinds of m6A modification patterns, which further understood the immune microenvironment of gastric cancer and provided the basis for precise treatment of gastric cancer.^36^ Therefore, we believe that this study is meaningful for the future precise treatment of severe asthma. Eosinophilic asthma is one of the important phenotypes of severe asthma.^37^ Eosinophil degranulation is involved in airway epithelial damage, and about 50%‐60% of asthma airway inflammation belongs to eosinophilic type.^38^, ^39^ Therefore, we also studied the relationship between eosinophil infiltration abundance and m6A regulator, and found that it is regulated by many m6A regulators, among which there is a significant positive correlation with YTHDF3 and a significant negative correlation with EIF3B. It was proved that these two regulators played an important role in the regulation of eosinophils in severe asthma. Finally, we identified the genes under the m6A modification patterns and key m6A regulators; the expression of these genes was affected by the m6A regulators. We found that two key genes of m6A and cluster 2 were closely related to turquoise module, which indicated that the expression of genes in this module was deeply affected by the m6A regulators. Therefore, we conducted functional enrichment analysis of these genes and found that they all affected biological processes, cell components, molecular functions and pathways in varying degrees, and then played an important role in severe asthma.

In conclusion, this secondary study from the U‐BIOPRED programme systematically analysed the relationship between m6A regulator modification and immune microenvironment. Combined with previous research methods^23^, ^36^, ^40^, ^41^, ^42^ and m6A detection technology‐merip‐seq, it has generated abundance results. Our study is the first one to analyse the m6A regulator‐mediated RNA methylation modification patterns and immune microenvironment infiltration characterization in severe asthma, which opens up a new direction for the research on the pathogenesis of immune‐related severe asthma with m6A modification mechanism, supplements the gap in the epigenetics of severe asthma, and encourages more scholars to carry out more research in this field. Nevertheless, all our findings confirmed the effect of m6A modification on the immune characteristics of severe asthma and provide new insights into the pathogenesis of severe asthma.

## CONFLICTS OF INTEREST

The authors confirm that there are no conflicts of interest.

## AUTHOR CONTRIBUTION

**Deyang Sun:** Conceptualization (lead); Formal analysis (equal); Software (equal); Validation (equal); Visualization (equal); Writing‐original draft (lead); Writing‐review & editing (lead). **Huan Yang:** Software (equal); Validation (equal); Visualization (equal); Writing‐original draft (equal); Writing‐review & editing (equal). **Liming Fan:** Data curation (equal). **Fenglin Shen:** Data curation (equal). **Zhen Wang:** Funding acquisition (supporting); Project administration (lead); Supervision (lead).

## Data Availability

The data that support the findings of our study are openly available in the Gene Expression Omnibus (GSE69683) at https://www.ncbi.nlm.nih.gov/geo/.
